# The Treatment of Syphilis with Report of Sixty-Five Cases Treated at Bellevue Hospital

**Published:** 1901-04

**Authors:** 


### THE TREATMENT OF SYPHILIS WITH REPORT OF SIXTY-FIVE CASES TREATED AT BELLEVUE HOSPITAL.

WINFIELD AYRES, New York, (*Philadelphia Med. Jour.*, November 10, 1900,) states that when his attention was called to mercurol as an antiseptic of special value in the treatment of gonorrhea, it occurred to him that it would be a first-class preparation for the treatment of syphilis. Some time was necessarily spent in determining the proper dosage. At first one-eighth of a grain was given three times daily, and this dose was gradually increased until it was found that three grains was the average quantity required to control the malady. The large amount given was seven grains and the smallest amount that exerted a controlling influence upon the disease was one-half grain. In starting a patient on a course of mercurol, the author advises beginning with half grain or grain doses. Salivation has been produced by two grains, and yet as much as six grains has been taken with no disagreeable symptoms.

The objections to the use of unguentum hydrargyri, as a remedy in secondary syphilis, are referred to and while the popularity of protoiodide of mercury is conceded, the irregularity of its action and its tendency to cause gastric and intestinal disturbances are not overlooked. In the writer's experience 33 per cent. of his cases were not benefited by this drug.

Mercurol is a nucleid of mercury, and was discovered by Karl Schwickerath, of Bonn, Germany. Kopp, Director of the Royal Polyclinic for Genito-urinary Diseases at the University of Munich, uses mercurol in smaller doses, which leads the writer to remark "he will find as I have done, that it is desirable to use a much larger dosage." Mercurol should not be given in solution with potassium iodide.

In all, 65 cases received mercurol at the Bellevue clinic, 60 of which had not had previous treatment. Of these, 13 did not return after the first or second visit; 14 did not remain long enough under treatment to give the preparation a fair trial, and 13 may be described as new patients. Deducting these 40 cases, there remain 25 cases that have been sufficiently long and regular in their attendance to supply data from which definite conclusions may be deducted. The detailed histories of these 25 cases are included in the paper. In summarising the author remarks that while two months' treatment of syphilis is insufficient to determine absolutely the value of any remedy, the marked improvement shown by many of his cases makes it certain that mercurol is of great value. Its superiority to mercuric chloride in controlling the symptoms of syphilis is proved. Like all internal remedies it has very little effect upon the initial lesion; still it has hastened the healing slightly. None of the cases required treatment with potassium iodide to control secondary manifestations.

To recapitulate: (1) mercurol causes less disturbance of the gastro-intestinal tract than any other preparation of mercury used internally; (2) it controls skin eruptions and pains much better than any other preparation, while it controls mucous eruptions as well as any other, and has equally as good an effect upon the chancre; (3) it is an advantage that it can be taken in pill form.

